# The Impact of Passive Leg Raising on Corrected Carotid Flow Time and Inferior Vena Cava Diameter Before and After Fasting: An Ultrasound‐Based Cross‐Sectional Study

**DOI:** 10.1002/hsr2.72621

**Published:** 2026-06-10

**Authors:** Iraj Golikhatir, Isa Nazar, Farzad Bozorgi, Freidoon Savadkoohi, Hamed Aminiahidashti

**Affiliations:** ^1^ Department of Emergency Medicine Mazandaran University of Medical Sciences Sari Iran; ^2^ Orthopedic Research Center Mazandaran University of Medical Sciences Sari Iran

**Keywords:** carotid arteries, fluid assessment, inferior vena cava, ultrasonography

## Abstract

**Background and Aim:**

Assessing the necessity for fluid resuscitation is critical in the emergency department, and sonography provides a less‐invasive, reproducible diagnostic modality for this purpose. This study aims to evaluate intravascular volume status in healthy individuals by analyzing alterations in corrected carotid flow time (FTc) and inferior vena cava (IVC) diameter via ultrasound before and after passive leg raising (PLR).

**Methods:**

This prospective observational study was conducted with healthy individuals who underwent a 12‐h fasting protocol. Participants voluntarily fasted, and we measured variations in blood pressure, heart rate, FTc, and IVC diameter before and after PLR in both fasting and non‐fasting conditions.

**Results:**

A total of 72 healthy male subjects were enrolled in the study. Statistically significant differences were observed in all measured parameters—blood pressure, heart rate, FTc, and IVC diameter—before and after PLR in both fasting and non‐fasting states (*p* < 0.001). Furthermore, a significant inversely correlation was identified between the increase in FTc and the reduction in IVC collapsibility following PLR (fasting: *p* = −0.567, *p* < 0.001; non‐fasting: *p* = −0.301, *p* = 0.010).

**Conclusion:**

FTc can be utilized as a reliable marker for assessing intravascular volume status, demonstrating a positive correlation with intravenous fluid administration. This index, when combined with other criteria for evaluating hemodynamic status, is effective for assessing patients' volume status.

## 1 Introduction

In the emergency department, accurate assessment of fluid responsiveness remains a major clinical challenge in clinical practice [1]. The volume of fluids administered varies significantly based on individual patient conditions, such as cardiac function and other factors influencing fluid tolerance [2]. The primary objective of fluid infusion is to augment cardiac output [3]; however, in the absence of appropriate fluid responsiveness assessment, only about 50% of patients demonstrate a meaningful increase in cardiac output following fluid bolus infusion [4]. Moreover, excessive fluid administration can elevate intravascular pressure and lead to edema [5]. Studies have indicated that limited fluid infusion may not adversely affect patient outcomes compared to unrestricted fluid administration [6]. Estimating cardiac preload is essential for assessing hydration levels in patients. Various methods exist for determining the fluid requirements of patients, and employing a combination of these approaches is recommended. The selection of a method for assessing fluid responsiveness largely depends on the patient's clinical status and underlying physiological conditions [7]. Ultrasonography, particularly through the measurement of inferior vena cava diameter (IVC‐D) and its variations during the respiratory cycle, serves as one criterion for fluid assessment. However, the reliability of IVC measurements can be compromised in patients with spontaneous breathing [8]. In contrast, corrected carotid flow time (FTc) has emerged as a promising predictor of fluid responsiveness, remaining unaffected by respiratory variations [9, 10]. The passive leg raising (PLR) maneuver is an effective strategy to augment cardiac preload, functioning as a self‐transfusion mechanism that can mobilize approximately 300 cc of blood into the circulatory system [11]. PLR induces larger increase in cardiac preload and may be used for predicting fluid [12]. This maneuver is independent of factors such as heart rate and breathing, making it reliable for patients with spontaneous breathing and cardiac arrhythmias [11]. This study aims to evaluate the utility of FTc and IVC‐D as noninvasive indicators of hydration status, specifically examining the effects of fasting (voluntary dehydration) on these parameters. Furthermore, we will investigate the influence of the PLR maneuver before and after fasting on FTc and IVC‐D in healthy subjects. By exploring these relationships, we hope to determine the applicability of these indicators in managing fluid administration for patients, thereby enhancing clinical decision‐making in fluid therapy.

## 2 Methods

### 2.1 Study Design and Setting

This prospective observational study was conducted at the Imam Khomeini hospital in Sari, north of Iran, involving participants without significant underlying diseases, including students, professors, and medical staff from the emergency department. Participants who voluntarily fasted for 12 h were included in the study.

### 2.2 Participants

Eligible participants were adults aged between 18 and 50 years who provided written informed consent. Exclusion criteria included the presence of any chronic or underlying diseases, fasting for less than 12 h, failure to undergo sonography after the fasting period, lack of a suitable sonographic view, and refusal to participate in the ultrasound assessment. A total of 72 participants were enrolled based on the following formula by considering a significance level (*α*) of 0.05, a power (1 − *β*) of 0.8, *d* = 0.70, and effect size σ=1.40 for DBP according to the study performed by Shokoohi et al. [13].

$$n=\frac{2*{\left(z_{1-\frac{\alpha }{2}}+z_{1-\beta }\right)}^{2}*\sigma^{2}}{d^{2}}.$$

### 2.3 Measurements

Upon enrollment, baseline blood pressure and heart rate were recorded while participants were in a supine position. Subsequently, ultrasound measurements were obtained using the Sonosite Edge II Ultrasound system (REF P23610‐10 SN Q2N7YP) with a 7.5 MHz linear probe, on the participants' beds. FTc and IVC‐D measurements of all participants in this study were performed only by the project administrator.

### 2.4 Carotid Artery Assessment

The carotid artery was first visualized in the horizontal plane, followed by measurements in the longitudinal plane. Spectral Doppler tracings were acquired from the center of the right carotid artery, positioned 2–3 cm proximal to the carotid bulb, aligned with the lower edge of the right thyroid lobe [14]. The angle of the ultrasound probe was adjusted to ensure it was parallel to the blood flow [15]. FTc was calculated using the formula [11]:

$$\text{FTc}=\frac{\text{Systole time}}{\sqrt{\text{Cycle time}}}.$$

Systole time was defined as the interval from the onset of the systolic upstroke to the beginning of the diastolic notch, while cycle time encompassed the complete cardiac cycle.

### 2.5 Inferior Vena Cava Assessment

The diameter of the IVC was measured 2 cm proximal to its entry into the right atrium, using a 5 MHz curved probe in M‐mode combined with 2D mode. Measurements were taken during both inhalation and exhalation. The IVC collapsibility index (cIVC) was calculated using the formula, where IVC max and IVC min represent the maximum diameter during expiration and the minimum diameter during inspiration, respectively [16].

$$\text{cIVC}=\frac{\text{IVC }\max-\text{IVC }\min}{\text{IVC }\max}.$$

### 2.6 Passive Leg Raising Maneuver

Following the initial measurements, participants were placed in a PLR position for 30–60 s. To perform PLR, each participant was first positioned at a 45° semi‐sitting angle, after which their legs were elevated to a 45° angle. The variables were then assessed within 30–60 s of this adjustment. This maneuver facilitates the movement of approximately 300 cc of blood from the lower limbs to the thorax, thereby increasing venous return (auto‐bolus) and enhancing the cardiac preload [17, 18, 19]. Clinical and sonographic variables were re‐evaluated during this period.

### 2.7 Post‐Fasting Assessment

After completing the 12‐h fasting period, participants entered the non‐fasting phase by consuming oral fluids without receiving intravenous fluids. All measurements were repeated 3 h later to assess changes in clinical and sonographic variables. In this study, the term “non‐fasting” refers specifically to this post‐fasting assessment phase.

### 2.8 Statistical Analysis

To summarize the continuous and qualitative variables, the mean ± SD and frequency (%) were applied, respectively. The normality assumption was evaluated using the Kolmogorov–Smirnov test. Then, the paired *T*‐test and Wilcoxon test were used to compare the mean of normal and non‐normal quantitative variables between before and after PLR and fasting status (Yes/No) as well, respectively. Then, the *q* values were computed using the FDR correction method. Assessing the relationships between two normal and non‐normal quantitative variables were performed using the Pearson or Spearman correlation coefficient tests, respectively. Furthermore, the multiple analysis of covariance (ANCOVA) regression model after adjusting the impact of potential confounders and pre‐PLR measurements was used to examine the effect of fasting status on the hemodynamic parameters after PLR including SBP, DBP, HR, systolic time, heart cycle, corrected FTc, IVC max, IVC min, and cIVC variables. All statistical analyses were performed in SPSS 20 at a two‐sided significance level of 0.05.

## 3 Results

**Figure 1:**
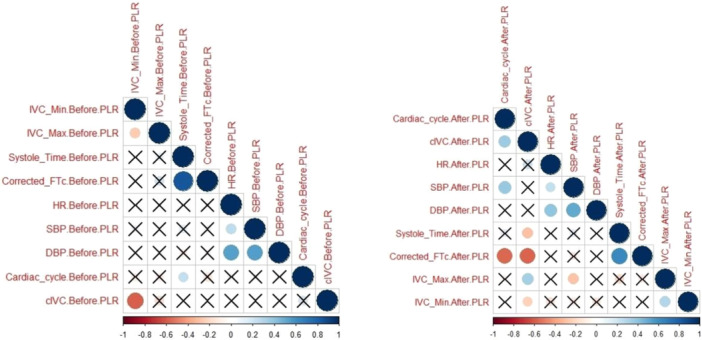
Correlation matrix between the hemodynamic parameters in fasting status pre‐PLR and post‐PLR. Negative and positive values indicate indirect and direct relationships, respectively; the insignificant value is specialized with a cross according to the significant level of 0.05.

**Figure 2:**
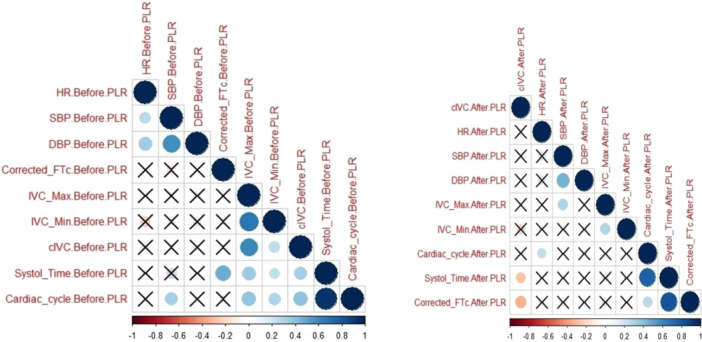
Correlation matrix between the hemodynamic parameters in non‐fasting status pre‐PLR and post‐PLR. Negative and positive values indicate indirect and direct relationships, respectively; the insignificant value is specialized with a cross according to the significant level of 0.05.

A total of 72 male participants (mean age: 23.90 ± 1.26 years, range: 22–26 years) were included in the study. The study population consisted exclusively of young, healthy male adults without known underlying diseases or comorbidities, yielding a relatively homogeneous baseline demographic profile. Hemodynamic parameters measured pre‐PLR and post‐PLR, stratified by fasting status, are summarized in Table 1. Analysis using the Wilcoxon test indicated significant differences in the median of all hemodynamic parameters between pre‐PLR and post‐PLR in the fasting group (*q* values < 0.05 for all). In the non‐fasting phase (3 h after completion of the 12‐h fasting period), all hemodynamic parameters showed statistically significant differences between pre‐PLR and post‐PLR regarding the median of hemodynamic parameters, except for IVC max (*q* values < 0.05 for all). Moreover, when considering both pre‐PLR and post‐PLR measurements, significant differences were observed in the median of SBP, DBP, HR, systolic time, IVC max, FTc, and cIVC between fasting and non‐fasting conditions at both time points (*q* values < 0.05 for all). Statistically significant differences in median cardiac cycle and IVC min were noted between fasting and non‐fasting conditions only at the pre‐PLR and post‐PLR assessments, respectively (*q* value < 0.05). Figures 1 and 2 illustrate the correlations between hemodynamic parameters in fasting and non‐fasting states pre‐PLR and post‐PLR.

**Table 1:** Comparison of the mean of hemodynamic parameters between pre‐PLR and post‐PLR in terms of fasting status.

Variables	Time	Status		*p*	*q*
		Fasting	Non‐fasting		
SBP^a^	Pre^b^	109.00^#^ [105.00–111.00]	113.50^#^ [110.00–117.00]	< 0.001*	< 0.001*
	Post^c^	110.50^#^ [107.25–115.00]	118.50^#^ [112.25–120.75]	< 0.001*	< 0.001*
*p*		< 0.001*	< 0.001*		
*q*		< 0.001*	< 0.001*		
DBP^d^	Pre	69.00 [65.00–71.00]^#^	70.50 [69.25–72.00]^#^	< 0.001*	< 0.001*
	Post	70.00 [67.00–72.00]^#^	72.00 [70.25–75.00]^#^	< 0.001*	< 0.001*
*p*		< 0.001*	< 0.001*		
*q*		< 0.001*	< 0.001*		
HR^e^	Pre	80.00 [79.00–82.75]^#^	75.00 [72.00–75.00]^#^	< 0.001*	< 0.001*
	Post	78.00 [75.00–79.00]	70.00 [68.25–71.00]	< 0.001*	< 0.001*
*p*		< 0.001*	< 0.001*		
*q*		< 0.001*	< 0.001*		
Systole time	Pre	492.50 [471.75–529.74]	552 [500.25–620.91]	< 0.001*	< 0.001*
	Post	501.16 [492.00–513.33]	599.00 [576.08–619.50]	< 0.001*	< 0.001*
*p*		0.04*	< 0.001*		
*q*		0.04*	< 0.001*		
Cardiac cycle	Pre	889.33 [872.08–906.33]	608.67 [577.83–839.91]	< 0.001*	< 0.001*
	Post	646.66 [609.58–701.75]	670.83 [627.00–692.91]	0.64	0.64
*p*		< 0.001*	0.01*		
*q*		< 0.001*	0.011*		
FTc^f^	Pre	16.87 [16.00–17.70]	21.58 [20.95–22.36]	< 0.001*	< 0.001*
	Post	19.68 [18.70–20.51]	23.19 [22.66–23.74]	< 0.001*	< 0.001*
*p*		< 0.001*	< 0.001*		
*q*		< 0.001*	< 0.001*		
IVC max^g^	Pre	1.13 [1.11–1.16]	0.94 [0.83–1.00]	< 0.001*	< 0.001*
	Post	1.02 [0.97–1.12]	0.92 [0.90–0.96]	< 0.001*	< 0.001*
*p*		< 0.001*	0.65		
*q*		< 0.001*	0.65		
IVC min^h^	Pre	0.66 [0.60–0.71]	0.67 [0.60–0.73]	0.07	0.074
	Post	0.69 [0.65–0.75]	0.75 [0.71–0.79]	< 0.001*	< 0.001*
*p*		< 0.001*	< 0.001*		
*q*		< 0.001*	< 0.001*		
cIVC^i^	Pre	43.50 [39.00–48.00]	27 [22.00–30.75]	< 0.001*	< 0.001*
	Post	33.00 [29.25–38.37]	18.20 [15.25–22.75]	< 0.001*	< 0.001*
*p*		< 0.001*	< 0.001*		
*q*		< 0.001*	< 0.001*		

SBP^a^: systolic blood pressure, Pre^b^: pre leg raising, Post^c^: post leg raising DBP^d^: diastolic blood pressure, HR^e^: heart rate, corrected FTc^f^: corrected carotid flow time, IVC max^g^: inferior vena cava maximum diameter, IVC min^h^: inferior vena cava minimum diameter, cIVC^i^: IVC collapsibility index.

^#^ Values are reported as median [IQR: Q1–Q3].

* Significant at the level of 0.05.

In the fasting state pre‐PLR, significant relationships were observed between cIVC and IVC min, cardiac cycle and systolic time, DBP and HR, SBP and HR, FTc and systolic time, as well as IVC max and IVC min (*p* < 0.05). In the fasting state post‐PLR, significant correlations were found between IVC min and cIVC, IVC max and cIVC, IVC max and SBP, FTc and parameters such as cardiac cycle and systolic time, and cIVC and cardiac cycle (*p* < 0.05). However, no significant relationships were identified between other variables in both measurement periods (*p* > 0.05).

In the fasting state pre‐PLR, significant correlations were also noted between the cardiac cycle and SBP, IVC min, IVC max, systolic time, and cIVC; between systolic time and FTc, IVC min, IVC max, and cIVC; and between DBP and SBP with HR (*p* < 0.05). In the non‐fasting state post‐PLR, significant relationships were observed between FTc and cIVC, cardiac cycle and systolic time, and IVC max with SBP (*p* < 0.05). However, other variables showed no significant relationships during both measurement periods (*p* > 0.05).

Table 2 presents the results of the multiple ANCOVA model. All ANCOVA assumptions were satisfied for the responses reported in Table 2. The model results indicated that all hemodynamic parameters, except for systolic time, cardiac cycle, and IVC max, significantly influenced the mean of hemodynamic parameters after PLR (*p* < 0.05). For instance after adjusting the effect of potential confounders, the means of SBP and DBP after PLR increased by 0.85 and 0.72 units, respectively, for each unit increase in SBP and DBP before PLR (*p* < 0.05). Furthermore after adjusting the effect of potential confounders, fasting status significantly affected the means of SBP (−1.16 [−1.87, −0.45]), DBP (−1.04 [−1.66, −0.42]), systolic time (−96.02 [−106.67, −85.37]), FTc (−2.78 [−3.64, −1.93]), IVC max (0.11 [0.06, 0.15]), IVC min (−0.03 [−0.06, −0.007]), and cIVC (2.83 [0.57, 5.09]) after PLR. In other words, these variables showed significant differences between fasting and non‐fasting statuses after PLR (*p* < 0.05). Specifically, after adjusting for potential confounders, the means of SBP and DBP after PLR in the fasting status were 1.16 and 1.04 units lower than in the non‐fasting status, respectively. However, fasting status did not significantly affect HR and cardiac cycle parameters after PLR.

**Table 2:** Comparison of the effect of fasting status on the response variables by controlling the effects of age and pre‐PLR measurements using the multiple ANCOVA model.

Response variables	Variables (reference)	Coefficients	%95 CI	*p*
SBP post‐PLR	SBP pre‐PLR	0.85	(0.78, 0.91)	< 0.001*
	Status (non‐fasting)			
	Fasting	−1.16	(−1.87, −0.45)	0.001*
DBP post‐PLR	DBP pre‐PLR	0.72	(0.65, 0.79)	< 0.001*
	Status (non‐fasting)			
	Fasting	−1.04	(−1.66, −0.42)	0.001*
HR post‐PLR	HR pre‐PLR	1.02	(0.95, 1.08)	< 0.001*
	Status (non‐fasting)			
	Fasting	0.34	(−0.28, 0.97)	0.27
Systole time post‐PLR	Systole time pre‐PLR	−0.04	(−0.13, 0.03)	0.28
	Status (non‐fasting)			
	Fasting	−96.02	(−106.67, −85.37)	< 0.001*
Cardiac cycle post‐PLR	Cardiac cycle pre‐PLR	−0.05	(−0.13, 0.02)	0.16
	Status (Non‐fasting)			
	Fasting	10.20	(−12.53, 32.94)	0.37
FTc post‐PLR	FTc pre‐PLR	0.18	(0.01, 0.34)	0.02*
	Status (non‐fasting)			
	Fasting	−2.78	(−3.64, −1.93)	< 0.001*
IVC max post‐PLR	IVC max pre‐PLR	0.04	(−0.11, 0.20)	0.57
	Status (non‐fasting)			
	Fasting	0.11	(0.06, 0.15)	< 0.001*
IVC min post‐PLR	IVC min pre‐PLR	0.55	(0.43, 0.66)	< 0.001*
	Status (non‐fasting)			
	Fasting	−0.03	(−0.06, −0.007)	0.01*
cIVC post‐PLR	cIVC pre‐PLR	0.70	(0.59, 0.81)	< 0.001*
	Status (non‐fasting)			
	Fasting	2.83	(0.57, 5.09)	0.01*

*Note:* Response variables = post‐PLR hemodynamic parameters measurement.

* Significant at the level of 0.05.

## 4 Discussion

This study demonstrated that FTc varied significantly between fasting and non‐fasting states, as well as before and after PLR. In the fasting state, we observed an inverse relationship between FTc and the cIVC following PLR. The main finding of this study is that corrected FTc differed significantly between fasting and non‐fasting states and increased significantly following PLR. Several studies have suggested that corrected FTc can effectively predict fluid requirements in patients, with measurements taken prior to fluid infusion serving as indicators of volume responsiveness in surgical patients [20]. However, other study has raised questions about the reliability of FTc in evaluating volume responsiveness [21]. In patients with septic shock, FTc has proven to be a valuable clinical tool for predicting fluid needs [22, 23]. Respiratory changes in the amplitude of the plethysmographic pulse wave (Delta P (PLET)) were shown to be accurate tools for predicting fluid responsiveness in septic patients [24]. Although some studies have shown a significant relationship between variation of pulse oximetric plethysmographic (POP) waveform amplitude (ΔPOP) and cardiac index (CI) after PLR in healthy participants, this relationship was too weak, so it cannot be used as a predictive tool for fluid responsiveness [25]. Another important finding of our study was the significant inverse relationship between changes in FTc and cIVC following PLR. In contrast, a study involving healthy subjects found no significant correlation between changes in cIVC and FTc following the Trendelenburg position [26]. Our findings, however, revealed a statistically significant relationship between increases in FTc and decreases in cIVC. This suggests that corrected FTc may serve as an alternative method for predicting changes in stroke volume (∆SV) in critically ill patients, particularly as transitioning from an upright to a head‐down position resulted in increases in corrected FTc, aligning with our results [27]. This relationship is significant because FTc is less invasive and can be easily performed at the bedside, aiding in fluid resuscitation—an increase in corrected FTc may indicate a need for fluid administration. In our study, the increase in FTc was statistically associated with reductions in cIVC after PLR in both fasting and non‐fasting states. Some studies have suggested that FTc can predict hypotension in surgical patients after induction [28]. However, in our investigation, despite significant increases in both systolic and diastolic blood pressure following PLR in the non‐fasting group, we found no correlation between blood pressure measurements and FTc. Despite significant changes in systolic and diastolic blood pressure after PLR, no significant correlation was observed between blood pressure parameters and FTc. Other studies have also reported no significant correlation between corrected FTc, mean arterial pressure (MAP), and pulse rate (PR) before and after PLR in critically ill patients in the intensive care unit, even though FTc accurately determined fluid responsiveness [29]. This discrepancy may be attributed to our study's focus on individuals with healthy cardiovascular systems, as other studies indicate that blood pressure measurements have limited sensitivity and specificity in assessing fluid responsiveness in healthy subjects [30]. Additionally, one study reported no statistically significant difference in corrected FTc between individuals experiencing spinal anesthesia‐induced hypotension (SAIH) and those with normal blood pressure, suggesting that corrected FTc may not be related to SAIH [31]. These findings suggest that FTc may serve as a practical, noninvasive bedside tool for assessing fluid responsiveness, particularly in settings where invasive monitoring is not feasible. The presence of this confounding factor, along with the higher average age of participants in that study compared to those in ours, may help explain these findings.

## 5 Limitations and Strengths

### 5.1 Strengths

This study has several strengths. A standardized and reproducible method was used, and both fasting and non‐fasting conditions were used. The effect of increased cardiac preload on FTc changes was assessed noninvasively using PLR. The use of a homogeneous population of healthy participants reduced confounding factors such as underlying diseases or medication use and increased the internal validity of the findings.

### 5.2 Limitation

This study was conducted exclusively with healthy participants, which may limit the generalizability of the findings to patient populations. Additionally, as a single‐centered investigation, the external validity of the results may be constrained. We faced challenges related to participant compliance; individuals who were unable or unwilling to continue in the study were excluded. Although fasting was implemented as a preparatory measure for testing, it did not pose significant risks to participants. Nonetheless, individuals at risk of hypotension or hypoglycemia were excluded after treatment for these complications to ensure their safety. Furthermore, the exclusive inclusion of male participants restricts the applicability of the findings to female populations.

## 6 Conclusion

The FTc can be used as a valuable tool for assessing fluid needs in individuals. Our findings indicate that increases in FTc during PLR and the transition from fasting to non‐fasting states reflect its significant influence on intravascular volume status of subjects. While FTc should not be relied upon in isolation for evaluating fluid volume and resuscitation processes, it can be effectively integrated with other assessment criteria. This approach enhances the overall evaluation of hemodynamic status in patients, owing to the simplicity and practicality of using FTc in clinical settings. However, given that this study was conducted exclusively in young healthy male participants, the clinical applicability of FTc in more diverse populations and critically ill patients requires further validation in future studies.

## Author Contributions

**Iraj Golikhatir:** writing – original draft. **Isa Nazar:** methodology, formal analysis, software. **Farzad Bozorgi:** writing – original draft. **Freidoon Savadkoohi:** writing – original draft, writing – review and editing, data curation, investigation. **Hamed Aminiahidashti:** writing – original draft, writing – review and editing, supervision, project administration, conceptualization, resources, funding acquisition. All authors have read and approved the final version of the manuscript.

## Disclosure

The lead author, Hamed Aminiahidashti, affirms that this manuscript is an honest, accurate, and transparent account of the study being reported; that no important aspects of the study have been omitted; and that any discrepancies from the study as planned (and, if relevant, registered) have been explained.

## Ethics Statement

The study protocol was approved by the Ethics Committee of Mazandaran University of Medical Sciences (Approval Code: IR.MAZUMS.IMAMHOSPITAL.REC.1399.5573).

## Consent

Informed consent was obtained from all participants, ensuring confidentiality and adherence to the principles outlined in the Helsinki Declaration.

## Conflicts of Interest

The authors declare no conflicts of interest.

## Data Availability

The data sets generated and/or analyzed during the current study are available from the corresponding author on reasonable request. Hamed Aminiahidashti had full access to all of the data in this study and takes complete responsibility for the integrity of the data and the accuracy of the data analysis.
